# Pyk2 uncouples metabotropic glutamate receptor G protein signaling but facilitates ERK1/2 activation

**DOI:** 10.1186/1756-6606-3-4

**Published:** 2010-01-21

**Authors:** Alexander A Nicodemo, Macarena Pampillo, Lucimar T Ferreira, Lianne B Dale, Tamara Cregan, Fabiola M Ribeiro, Stephen SG Ferguson

**Affiliations:** 1 J Allyn Taylor Centre for Cell Biology, Molecular Brain Research Group, Robarts Research Institute, The University of Western Ontario, 100 Perth Dr, London, ON, N6A 5K8, Canada; 2 Department of Physiology & Pharmacology, The University of Western Ontario, 100 Perth Dr, London, ON, N6A 5K8, Canada

## Abstract

Group I metabotropic glutamate receptors (mGluRs) are coupled via Gα_q/11 _to the activation of phospholipase Cβ, which hydrolyzes membrane phospholipids to form inositol 1,4,5 trisphosphate and diacylglycerol. This results in the release of Ca^2+ ^from intracellular stores and the activation of protein kinase C. The activation of Group I mGluRs also results in ERK1/2 phosphorylation. We show here, that the proline-rich tyrosine kinase 2 (Pyk2) interacts with both mGluR1 and mGluR5 and is precipitated with both receptors from rat brain. Pyk2 also interacts with GST-fusion proteins corresponding to the second intracellular loop and the distal carboxyl-terminal tail domains of mGluR1a. Pyk2 colocalizes with mGluR1a at the plasma membrane in human embryonic kidney (HEK293) cells and with endogenous mGluR5 in cortical neurons. Pyk2 overexpression in HEK293 results in attenuated basal and agonist-stimulated inositol phosphate formation in mGluR1 expressing cells and involves a mechanism whereby Pyk2 displaces Gα_q/11 _from the receptor. The activation of endogenous mGluR1 in primary mouse cortical neuron stimulates ERK1/2 phosphorylation. Treatments that prevent Pyk2 phosphorylation in cortical neurons, and the overexpression of Pyk2 dominant-negative and catalytically inactive Pyk2 mutants in HEK293 cells, prevent ERK1/2 phosphorylation. The Pyk2 mediated activation of ERK1/2 phosphorylation is also Src-, calmodulin- and protein kinase C-dependent. Our data reveal that Pyk2 couples the activation mGluRs to the mitogen-activated protein kinase pathway even though it attenuates mGluR1-dependent G protein signaling.

## Background

Glutamate is the predominant excitatory neurotransmitter in the brain and regulates physiological responses that include the development, survival, and death of neurons as well as neural plasticity [1-6]. The receptors that respond to glutamate can be classified into two types: ionotropic and metabotropic. Ionotropic glutamate receptors are comprised of cation-specific ion channels that can be subclassified into NMDA, AMPA and kainate receptors. In contrast, metabotropic glutamate receptors (mGluRs) are coupled to the activation of heterotrimeric G proteins and the regulation of intracellular second messenger levels. They are subcategorized into three groups based on sequence similarity and G protein specificity [1]. Group I mGluRs (mGluR1 and mGluR5) are coupled to Gα_q/11 _and the activation of phospholipase Cβ which in turn stimulates the formation of the intracellular second messengers, inositol 1,4,5-triphosphate (InsP_3_) and diacylglycerol. InsP_3 _stimulates the release of Ca^2+ ^from intracellular stores and both Ca^2+ ^and diacylglycerol regulate the activation of protein kinase C (PKC).

Agonist-activated G protein-coupled receptors (GPCRs) function as guanine nucleotide exchange factors facilitating the exchange of GDP for GTP on the Gα subunit. Thus, allowing the dissociation of the Gα and Gβγ subunits, which function to regulate different effector enzymes and ion channels [7]. The activity of Group I mGluRs is regulated by protein kinases that uncouple the receptors from heterotrimeric G proteins via both phosphorylation-dependent- and phosphorylation-independent mechanisms [8,9]. Both PKC and G protein-coupled receptor kinase 4 (GRK4) uncouple Group I mGluR signaling by phosphorylating serine and threonine residues localized to the intracellular loop and carboxyl terminal tail domains of the receptors [10-14]. In contrast, the attenuation of both mGluR1 and mGluR5 signaling by GRK2 is mediated by a phosphorylation-independent mechanism that involves the interaction of the GRK2 amino terminal regulator of G protein signaling homology domain with Gα_q/11 _resulting the displacement of the heterotrimeric G protein from the receptor [9,15]. Recently, we have demonstrated that optineurin and calcineurin inhibitory protein (CAIN) can also contribute to the phosphorylation-independent attenuation of Group I mGluR signaling by a mechanism that also involves Gα_q/11 _displacement from the receptor [16,17].

A number of studies have also implicated tyrosine kinases in the regulation of Group I mGluR signaling. For example, Canepari *et al *[18] have reported that inhibitors of tyrosine phosphorylation enhance mGluR1-mediated cation-permeable ion channel conductances at parallel fibers that synapse on Purkinje neurons in the cerebellum of the rat. In contrast, G protein-dependent inward currents in midbrain dopaminergic neurons activated by mGluR1 are antagonized by tyrosine kinase inhibitors [19]. A potential candidate tyrosine kinase that is involved in the regulation of Group I mGluR signaling is proline-rich tyrosine kinase 2 (Pyk2), which is found in a multimolecular complex with both mGluR1 and the NMDA receptor [20]. Heidinger and coworkers [21] have also demonstrated that mGluR1-mediated upregulation of NMDA receptor currents in cortical neurons involves a Ca^2+^-, calmodulin- and Src-dependent activation of Pyk2. We show here that Pyk2 can be co-immunoprecipitated with both mGluR1 and mGluR5 from rat brain lysates and that Pyk2 interacts directly with Group I mGluRs via their second intracellular loop and carboxyl-terminal tail domains. We also find that Pyk2 expression attenuates mGluR1a signaling by disrupting receptor/Gα_q/11 _interactions, but functions to couple mGluR1 to the activation of extracellular regulated kinase 1/2 (ERK1/2) in cortical neurons.

## Methods

### Materials

Human embryonic kidney (HEK293) cells and COS-7 cells were obtained from the American Type Culture Collection (Manassas, VA, USA). Tissue culture reagents were purchased from Invitrogen (Burlington, ON, Canada). Isopropyl-β-D-thiogalactopyranoside (IPTG) was obtained from Bioshop Canada Inc (Mississauga, ON, Canada). Quisqualate, DHPG, MPEP and LY367385 were purchased from Tocris Cookson Inc. (Ellisville, MO, USA). [^3^H]myo-inositol was acquired from PerkinElmer Life Sciences (Woodbridge, ON). The Dowex 1-X8 (formate form) resin with 200-400 mesh was purchased from Bio-Rad. Horseradish peroxidase-conjugated anti-mouse and anti-rabbit IgG secondary antibodies, protein G-Sepharose beads, enhanced chemiluminescence (ECL), and mouse monoclonal anti-hemagglutinin (HA) (12CA5) antibody were purchased from GE Healthcare (Oakville, ON Canada). Anti-GST mouse monoclonal antibody and anti-G_αq/11 _rabbit polyclonal antibody were from Santa Cruz Biotechnology Inc. (Santa Cruz, CA, USA). Anti-mGluR5 rabbit polyclonal antibody was obtained from Upstate (Lake Placid, NY, USA). Anti Pyk2/CAKβ mouse monoclonal antibody was from BD Transduction Laboratories (Mississauga, ON, Canada). Rabbit polyclonal phospho-p44/44 MAP kinase (Thr202/Tyr402), p44/44 MAP kinase, phospho-Pyk2 (Tyr402) antibody, phospho-Pyk2 (Tyr457), Pyk2, phosphor-Src (Tyr 416) and Src antibodies were all obtained from Cell Signaling Technology. (Pickering, ON, Canada). Lipofectamine Alexa Fluor 488 goat anti-mouse IgG and Alexa Fluor 568 goat anti-rabbit IgG were purchased from Invitrogen/Molecular Probes (Burlington, ON, Canada). siRNAs were purchased from Dharmacon RNAi Technologies (Lafayette, CO, USA). Hi-Perfect Transfection Reagent was purchased from Qiagen (Misssauga, ON, Canada). All other biochemical reagents were purchased from Sigma (Oakville, ON, Canada), Fisher Scientific (Ottawa, ON, Canada) and VWR Scientific (Mississauga, ON, Canada).

### Plasmid construction

The cDNA for the human Pyk2 was first amplified by PCR from the human universal Quick Clone™ library and tagged with a hemagglutanin (HA) epitope tag (BD Biosciences/Clontech, Mississauga, ON, Canada). The PCR product generated was digested with *SalI/XbaI *and subcloned into a mammalian expression HA vector. All other cDNA constructs have been described previously [12,17].

### Cell Culture and transfection

HEK293 cells were maintained in Eagle's minimal essential medium supplemented with fetal bovine serum (8% v/v) and gentamicin (5 μg/ml). African green monkey (COS7) cells were grown at 37°C in Dulbecco's modified Eagle's medium supplemented with fetal bovine serum (8% v/v) and gentamicin (5 μg/ml). Cells were transfected using a modified calcium phosphate method as described previously [22]. Following transfection (18 h), the cells were incubated with fresh medium and allowed to recover for 24 h for coimmunoprecipitation studies. Otherwise, they were allowed to recover for 6-8 h and reseeded into either 12-well dishes coated with collagen or containing collagen-coated coverslips, or into 24-well dishes and then grown an additional 18 h prior to experimentation.

Primary neuronal cultures were prepared from the cortex of embryonic day 15 CD1 mouse embryos. Cells were plated on either 100 mm dishes or 15 mm glass coverslips coated with poly-L-ornithine (Invitrogen) in Neurobasal media with B-27 (Gibco) and N2 (Gibco) supplements, 2 mM glutamax, 50 μg/ml penicillin, 50 mg/ml streptomycin. Cells were incubated at 37°C and 5% CO_2 _in a humidified incubator and cultured for up to 21 days *in vitro *with media replenishment every 3 days. Rat primary cortical cultures were cultured as described for primary mouse cultures and were prepared from the cortex of embryonic day 20 Wistar rat embryos. All animal procedures were approved by the University of Western Ontario Animal Care Committee.

### siRNA Transfection

After a growth period of 4 days, primary mouse cortical neurons growing in 6-well or 12-well plates were transfected with 100 nM of Pyk2 siRNA (GAACATGGCTGACCTCATATT) using Hi-Perfect Transfection Reagent according to the manuscfacturer's specifications (Qiagen, Mississauga, ON). After two days, the cells were transfected again in the exact same manner. The next day, the neurobasal media was aspirated, and the cells were washed twice with HBSS and left overnight in HBSS supplemented with bovine serum albumin Fraction V. For COS7 cells Pyk2 siRNA (GGACGAGGACTATTACAAA) was transfected with Lipofectamine 2000 following manufacturer's instructions. Scrambled siRNA (Non-Targeting siRNA #1) was purchased from Dharmacon. Experiments were performed 48 or 72 hours after transfection and knock down of proteins was confirmed by western blot.

### Co-immunoprecipitation

Coimmunoprecipitation experiments were performed using 500-1000 μg of total cell lysate protein solubilized from HEK293 cells transiently transfected with the various cDNA constructs as described in the *Figure Legends*. The cells were solubilized in lysis buffer (25 mM HEPES, pH 7.5, 300 mM NaCl, 1.5 mM MgCl2, 0.2 mM EDTA and 0.1% Triton X-100) containing protease inhibitors. Phosphorylated Pyk2 was examined in the presence of 1 mM sodium vanadate, 100 μM GDP, 2 mM MgSO4, 30 μM aluminum chloride, and 5 mM sudium fluoride. FLAG-mGluR1a was immunoprecipitated with FLAG Sepharose beads from cell lysates by overnight rotation at 4°C. For the coimmunoprecipitation of endogenous proteins from whole-brain extracts, adult rat brains were employed. Tissue was dissected and homogenized on ice in lysis buffer containing protease inhibitors. The particulate fraction was removed by centrifugation and 500-1000 μg of supernatant protein was incubated with polyclonal anti-mGluR1a or anti-mGluR5a rabbit antibodies and protein G-sepharose beads by overnight rotation at 4°C. For HEK293 and rat brain co-immunoprecipitations, the beads were washed 4-6 times with lysis buffer, and proteins were eluted in SDS loading buffer by boiling for 3-5 min. Eluted samples were separated by SDS-PAGE, followed by electroblotting onto nitrocellulose membranes for immunoblotting. Immunoblots were visualized by chemiluminescence using an ECL kit. Intensities of immunoblot signals were determined with the 4.0.2 version of the data acquisition and analysis software from Scion Corp. (Frederick, MD, USA).

### Confocal microscopy and Immunostaining

Confocal microscopy was performed using a Zeiss LSM-510 META laser scanning confocal microscope equipped with a Zeiss 63X, 1.4 numerical aperture, oil immersion lens (North York, ON, Canada). HEK293 cells expressing both HA-Pyk2 and FLAG-mGluR1a as well as primary rat cortical neurons were seeded on 15 mm glass coverslips. HEK293 cells were serum starved for 30 min at 37°C in HBSS (116 mM NaCl, 20 mM HEPES, 11 mM glucose, 5 mM NaHCO3, 4.7 mM KCl, 2.5 mM CaCl2, 1.2 mM MgSO4, 1.2 mM KH2PO4, pH 7.4). HEK293 cells were prelabeled with Alexa Fluor 568-conjugated anti-FLAG polyclonal rabbit antibody. Cells were then either kept on ice or stimulated with 100 μM quisqualate for 30 min at 37°C. Subsequently, the cells were fixed with 4% paraformaldehyde and permeabilized with 0.05% Triton X-100, and labeled with Alexa Fluor 568-conjugated anti-HA mouse monoclonal antibody. Primary rat cortical neurons were fixed with 4% paraformaldehyde and permeabilized with 0.05% Triton X-100, and then stained for endogenous mGluR5a and Pyk2 with rabbit polyclonal anti-mGluR5a antibody and mouse monoclonal anti-Pyk2/CAKβ antibody. Coverslips were mounted in IMMU-MOUNT (Thermo Shandon, Pittsburgh, PA) onto glass slides and allowed to air dry before viewing. Colocalization studies were performed using dual excitation (488, 543 nm) and emission (band pass 505-530 nm and long pass 560 nm for Alexa Fluor 488 and 568, respectively) filter sets. The specificity of labeling and absence of signal crossover were established by examination of single-labeled samples.

### GST-mGluR1a fusion protein purification and pull down assays

GST-mGluR1a peptides were generated by growing recombinant *Escherichia coli *BL21 bacteria at 37°C to an A_600 _of 0.65-0.8. Bacterial cultures were then induced for 3 hrs with 1 mM IPTG. After induction, cultures were pelleted, resuspended in PBS containing protease inhibitors and lysed by mild sonication. The bacterial lysates were cleared of cellular debris by centrifugation and then incubated with to glutathione-Sepharose 4B resin overnight at 4°C. GST-mGluR1a peptides bound to the matrix were washed extensively in PBS and then resuspended in lysis buffer containing protease inhibitors. COS-7 cell lysates overexpressing HA-Pyk2 were prepared and cleared of cellular debris by centrifugation. 400 μg of total cell lysate protein was used in each pull-down assay. One μg of matrix-bound mGluR1a peptides were incubated together with COS7 cell lysates and mixed overnight on an orbital shaker at 4°C. The matrix-bound protein complexes were washed extensively in lysis buffer and then eluted off the matrix in SDS loading buffer by boiling for 3-5 mins. Eluted samples were analyzed by SDS-PAGE and Western blotting with anti-Pyk2.

### Inositol Phosphate Formation

Inositol lipids were radiolabeled by incubating HEK293 cells overnight with 1 μCi/ml [^3^H]myo-inositol in Dulbecco's modified Eagle's medium. Unincorporated [^3^H]myo-inositol was removed by washing the HEK293 cells with HBSS. HEK293 cells were preincubated for 1 h in HBSS at 37°C and then preincubated in 500 μl of the same buffer containing 10 mM LiCl for an additional 10 min at 37°C. HEK293 cells were subsequently treated in either the absence or the presence of increasing concentrations (0-30 μM) of quisqualate for 30 min at 37°C. The reaction was stopped on ice by adding 500 μl of 0.8 M perchloric acid and then neutralized with 400 μl of 0.72 M KOH, 0.6 M KHCO3. The total [^3^H]myo-inositol incorporated into the cells was determined by counting the radioactivity present in 50 μl of the cell lysate. Total inositol phosphate was purified from the cell extracts by anion exchange chromatography using Dowex 1-X8 (formate form) 200-400 mesh anion exchange resin. [^3^H]Inositol phosphate formation was determined by liquid scintillation using a Beckman LS 6500 scintillation system.

### Receptor internalization

Agonist-independent FLAG-mGluR1a internalization was measured by flow cytrometry. Cell surface epitope-tagged receptors were prelabeled with primary anti-mouse FLAG antibody (1:500) on ice for 45 min. Cells were then warmed to 37°C in the absence of agonist for the times indicated in the *Figure Legends*. Cells were then transferred back on ice and labeled with secondary FITC-conjugated anti-mouse IgG antibody (1:500 dilution) for 45 min. Under these conditions, receptors are able to undergo only a single round of internalization. Receptor internalization is defined as the fraction of total cell receptors lost from the cell surface and thus is not available for labeling with the secondary antibody.

### ERK1/2 and Pyk2 immunoblots

100 μg samples of cell lysate proteins solubilized from either HEK293 cells, COS7 cells or mouse primary cortical neurons were subjected to were subjected to SDS-PAGE, followed by electroblotting onto nitrocellulose membranes for immunoblotting with antibodies described in the *Figure Legends*. The primary antibodies used in the experiments were phospho-p44/44 MAP kinase (Thr202/Tyr402) antibody (diluted 1:1000), p44/44 MAP kinase antibody (1:1000), phospho-Pyk2 (Tyr402) antibody (1:1000), phospho-Pyk2 (Tyr457) antibody (1:1000), Pyk2 antibody (1:1000), rabbit anti-Flag antibody (1:1000). Antibody reactive bands were visualized and quantified as described above.

### Data Analysis

The means ± S.E.M. are shown for values obtained for the number of independent experiments indicated in the figure legends. GraphPad Prism software (Graph Pad, San Diego, CA) was used to analyze data for statistical significance, as well as to analyze and fit dose-response and time course data. The statistical significance was determined by either one way analysis of variance or t-test.

## Results

### Interaction between Pyk2 and mGluR1

**Figure 1:**
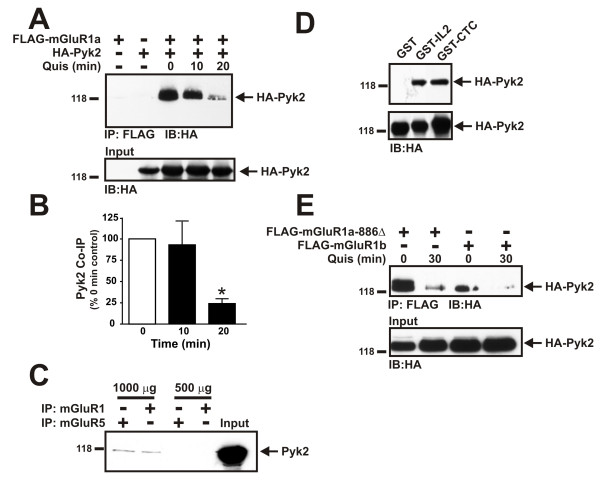
**Pyk2 interacts with Group I mGluRs**. **A) **Representative immunoblot showing the co-immunoprecipitation of HA-Pyk2 with Flag-mGluR1a from HEK293 treated with 30 μM quisqualate for 0, 10, and 20 min. FLAG-mGluR1a is immunopreciptated from 500-1000 μg of protein lysate. The expression of HA-Pyk2 is shown in input lanes correspond to 100 μg of protein lysate. **B) **The densitometric analysis of autoradiographs showing the mean ± SD of 5 independent experiments examining the co-immunoprecipitation of HA-Pyk2 with FLAG-mGluR5. * P < 0.05 versus untreated cells. **C) **Representative immunoblotblot showing the co-immunoprecipitation of Pyk2 with mGluR1 and mGluR5 polyclonal antibodies from either 1000 or 500 μg of rat whole brain lysates. The blot is representative of 3 independent experiments. **D) **Representative immunoblot of HA-Pyk2 co-precipitated with 1 μg of GST, and GST fusion proteins encoding either the second intracellular loop domain of mGluR1 (GST-IL2) or the distal region of the carboxyl-terminal tail domain of mGluR1a (GST-CTC). The GST fusions are incubated with 400 μg of COS-7 cell lysates expressing HA-Pyk2. The expression of HA-Pyk2 is shown in input lanes correspond to 100 μg of protein lysate. **E) **Representative immunoblot showing the co-immunoprecipitation of HA-Pyk2 with either Flag-mGluR1a-866Δ or FLAG-mGluR1b and HA-Pyk2 and treated with 30 μM quisqualate for 0 and 30 min. FLAG-mGluR1 protein is immunopreciptated from 500-1000 μg of protein lysate. The expression of HA-Pyk2 is shown in input lanes correspond to 100 μg of protein lysate. Data are representative of 4 independent experiments.

It was previously reported that both Pyk2 and mGluR1a were components of a protein complex that could be co-immunoprecipitated with the NMDA receptor [20]. Moreover, it was shown that mGluR1a activation altered NMDA receptor currents in a Pyk2-dependent manner [21]. Therefore, we tested whether HA-Pyk2 could be co-immunoprecipitated from HEK293 cells with FLAG-mGluR1a. We found that HA-Pyk2 was co-immunoprecipitated with FLAG-mGluR1 in the absence of agonist and agonist treatment for 20 min reduced the amount of HA-Pyk2 co-immunoprecipitated with FLAG-mGluR1a to 24 ± 6% of control (Fig. 1A and 1B). We also found that endogenous Pyk2 could be co-immunoprecipitated with either mGluR1 or mGluR5 from rat whole brain lysates (Fig. 1C). To assess whether HA-Pyk2 interacted directly with mGluR1a, we tested the ability of GST fusion proteins encoding either the second intracellular loop (IL2) or carboxyl terminal tail (CTC) domains of mGluR1a to co-precipitate HA-Pyk2 from COS7 cell lysates. We found that HA-Pyk2 bound to both the GST-IL2 and GST-CTC fusion proteins (Fig. 1D). HA-Pyk2 could be co-immunoprecipitated with both a mGluR1a mutant (mGluR1a-866Δ) and the mGluR1b splice variant that lack the extended carboxyl-terminal tails and the association of HA-Pyk2 was decreased following agonist treatment (Fig. 1E). Taken together, these results indicated that Pyk2 forms a complex with Group I mGluRs by binding to the IL2 and carboxyl terminal tail domains of the receptors, but that the distal portion of the mGluR1a carboxyl-terminal tails was not essential for Pyk2 interactions with the receptor.

### Colocalization of Pyk2 and mGluR1

**Figure 2:**
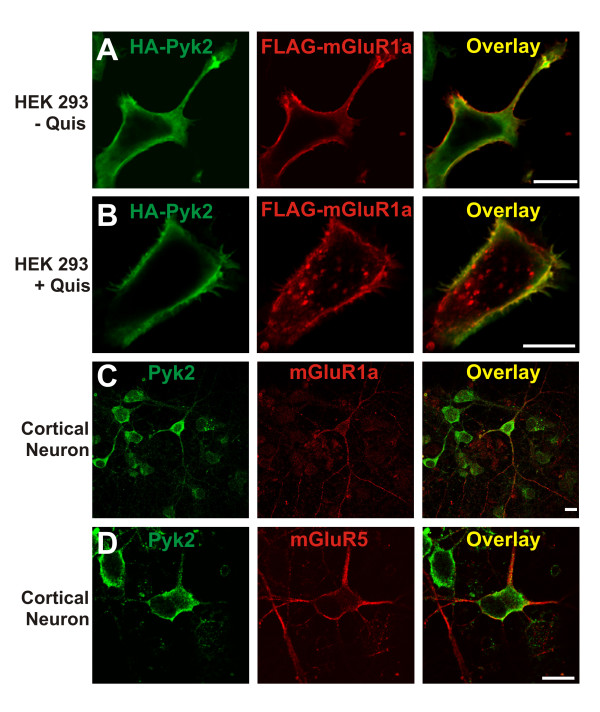
**Pyk2 colocalizes with Group I mGluRs in HEK 293 cells and in primary mouse cortical neurons**. Shown are representative confocal micrographic images of HEK293 cells showing the distribution of HA-Pyk2 and FLAG-mGluR1a distribution in either **A) **the absence of agonist treatment or **B) **following treatment with 100 μM quisqualate for 30 min. Cells are transfected with 1 μg of pcDNA3.1 plasmid cDNA encoding HA-Pyk2 and 5 μg of pcDNA3.1 plasmid cDNA encoding FLAG-mGluR1 and are stained with Alexa Fluor-conjugated 568 rabbit polyclonal anti-Flag antibody and Alexa Fluor 488-conjugated mouse monoclonal anti-HA antibody at 4°C. The data is representative 3 independent experiments. Shown are representative confocal micrographic images of primary rat cortical neurons fixed in 4% paraformaldehyde and stained with either Alexa Flour 568-conjugated rabbit polyclonal anti-mGluR1a **(C) **or mGluR5 **(D) **antibody and Alexa Fluor 488-conjugated polyclonal rabbit anti-Pyk2 antibody. The micrograph is representative of 3 independent experiments. Scale bars, 10 μm.

**Figure 3:**
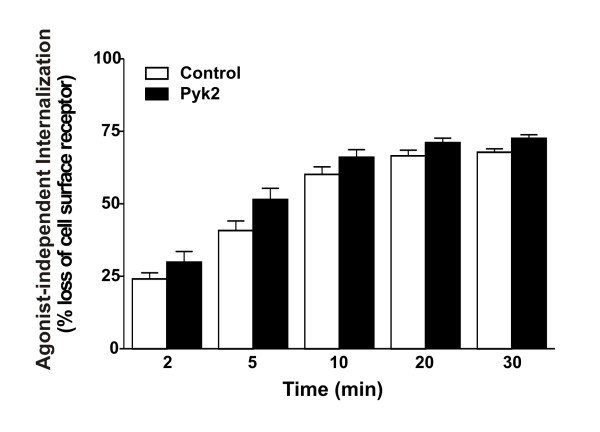
**Pyk2 expression has no effect on agonist-independent receptor internalization**. HEK293 cells transfected with 5 μg of pcDNA3.1 cDNA encoding FLAG-mGluR1a with and without either 5 μg of empty pcDNA3.1 plasmid cDNA or 5 μg of pcDNA3.1 plasmid cDNA encoding HA-Pyk2 were treated with and without 100 μM quisqualate for different time points. The data represent the mean ± S.E.M. of 9 independent experiments.

Because Pyk2 can be co-immunoprecipitated with mGluR1a, we examined whether Pyk2 was colocalized with Group I mGluRs in both HEK293 cells and primary rat cortical neurons. HEK293 cells expressing FLAG-mGluR1a and HA-Pyk2 were labeled on ice with Alexa Fluor 568-congugated FLAG antibody and treated either with or without 100 μM quisqualate for 45 min at 37°C. Subsequently, the cells were fixed and permeabilized and labeled with Alexa Fluor 488-conjugated HA antibody to stain HA-Pyk2. In the absence of agonist stimulation, FLAG-mGluR1a was localized to the cell surface and although HA-Pyk2 was expressed throughout the cytoplasm, Pyk2 was also localized at the plasma membrane (Fig. 2A). In response to agonist treatment, cell surface FLAG-mGluR1a was internalized to intracellular vesicles, but HA-Pyk2 was not observed to internalize as a complex with the receptor (Fig. 2B). We also found that primary rat cortical neurons, which stained positive for either endogenous mGluR1a or mGluR5 expression, also stained positive for the expression of Pyk2 protein (Fig. 2C and 2D). Consistent with the observation that HA-Pyk2 does not internalize with FLAG-mGluR1a (Fig. 2B), HA-Pyk2 overexpression did not alter the extent of agonist-independent FLAG-mGluR1a internalization (Fig. 3).

### Pyk2-mediated attenuation of mGluR1a signaling

**Figure 4:**
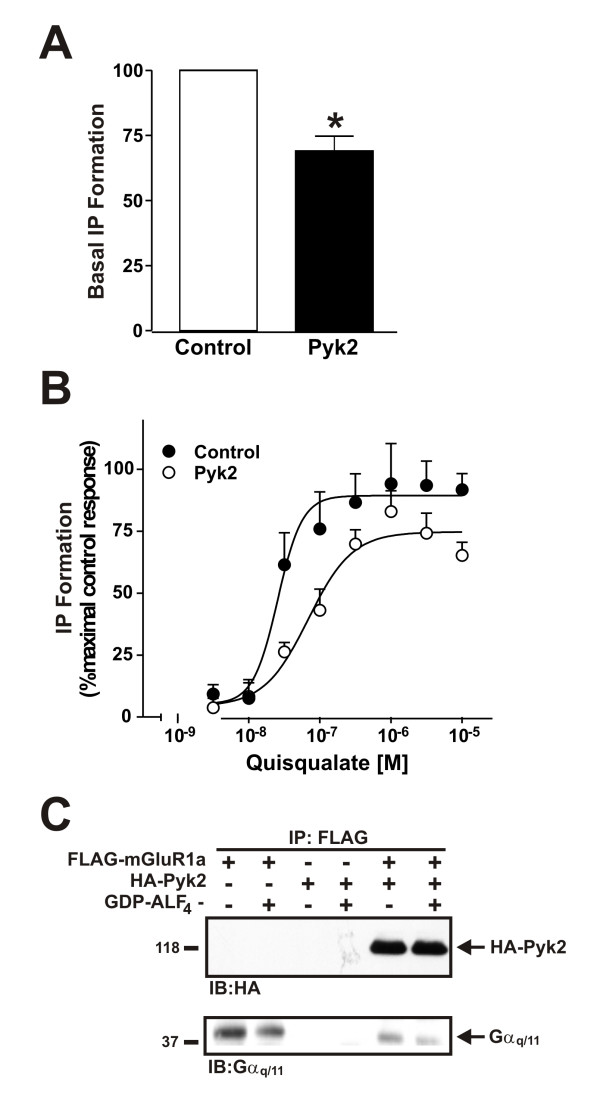
**Effect of Pyk2 expression on mGluR1a signaling**. HEK293 cells transfected with 5 μg of pcDNA3.1 cDNA encoding FLAG-mGluR1a with and without either 5 μg of empty pcDNA3.1 plasmid cDNA or 5 μg of pcDNA3.1 plasmid cDNA encoding HA-Pyk2 were assessed for IP formation in either **A) **the absence of agonist stimulation or **B) **in response to increasing concentrations of quisqualate (10^-8.5^-10^-4.5^M). The agonist dose-response curves were fit and analyzed using GraphPad Prism software. The data points represent the mean ± SEM for 10 independent experiments. *P < 0.05 versus control cells. **C) **Representative immunoblot showing the effect of HA-Pyk2 expression and GDP-ALF_4- _treatment on Gα_q/11 _co-immunoprecipitation with FLAG-mGluR1a. The data points represent the mean ± SD for 4 independent experiments. * P < 0.05 versus untreated cells.

Since we established that Pyk2 interacted with mGluR1, we tested whether this association affected mGluR1a-mediated inositol phosphate (IP) formation in HEK293 cells expressing FLAG-mGluR1a. We found that in the absence of agonist-treatment HA-Pyk2 reduced basal IP formation in FLAG-mGluR1a expressing HEK293 cells to 69 ± 6% of control values (Fig. 4A). Similarly, the overexpression of HA-Pyk2 reduced the maximal FLAG-mGluR1a-mediated responses to increasing concentrations of quisqualate to 75 ± 7% of control values and induced a 2.9 fold rightward shift in the EC_50 _for quisqualate-stimulated IP formation (EC_50 _= 26 nM vs 76 nM) (Fig. 4B). Previously, we demonstrated that both GRK2 and CAIN attenuated mGluR1a signaling by competing with Gα_q/11 _for binding to IL2 of mGluR1a [9,17]. Therefore, we examined whether the overexpression of HA-Pyk2 also disrupted Gα_q/11 _interactions with FLAG-mGluR1a. In cells expressing HA-Pyk2, HA-Pyk2 was co-immunoprecipitated with FLAG-mGluR1a and resulted in a decrease in Gα_q/11 _co-immunoprecipitated with the receptor complex (Fig. 4C). The receptor Gα_q/11 _complex was also regulated in both the presence and absence of HA-Pyk2 by GDP-ALF_4-_, which binds to Gα_q/11 _and mimics the Gα_q/11 _transition state (Fig. 4C). Moreover, we were unable to demonstrate that Pyk2 overexpression led to mGluR1a tyrosine phosphorylation (data not shown). Thus, taken together, these results demonstrated that Pyk2 contributed to the regulation of mGluR1a signaling by antagonizing receptor interactions with Gα_q/11_.

### mGluR1 mediates ERK1/2 phosphorylation by a Src/Pyk2-dependent pathway

**Figure 5:**
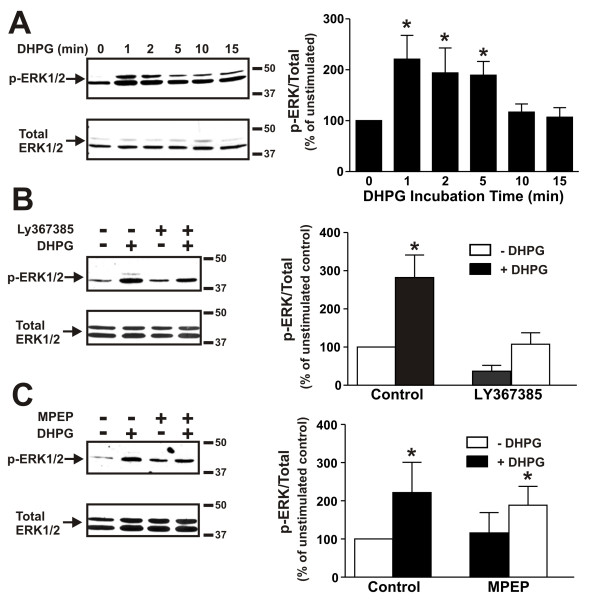
**ERK1/2 phosphorylation in mouse cortical neurons is mGluR1-dependent**. **A) **Representative immunoblot of the time course for ERK1/2 phosphorylation in primary mouse cortical neurons treated with 50 μM DHPG. Bar graph shows the denstiometric analysis of the time course of DHPG-stimulated ERK1/2 phosphorylation in cortical neurons. The data represents the mean ± SD of four independent experiments. * P < 0.05 versus untreated cells. **B) **Representative immunoblot showing the effect of treating primary mouse cortical neurons with and without 50 μM DHPG for 5 min following pretreatment either with or without the mGluR1-selective antagonist LY367385 (100 μM) on ERK1/2 phosphorylation. Bar graph shows the mean ± SD of five independent experiments. **C) **Representative immunoblot showing the effect of treating primary mouse cortical neurons with and without 50 μM DHPG for 5 min following pretreatment either with or without the mGluR5-selective antagonist MPEP (10 μM) on ERK1/2 phosphorylation. Bar graph shows the mean ± SD of four independent experiments. * P < 0.05 versus untreated mGluR1a expressing cells.

A previous study demonstrated that mGluR1 regulated NMDA receptor currents via the activation of a Pyk2/Src-family kinase pathway [21]. Therefore, we examined whether Pyk2 regulated mGluR1-mediated ERK1/2 phosphorylation in addition to heterotrimeric G protein coupling. The treatment of primary mouse cortical neurons with 50 μM (S)-3,5-dihydroxylphenylglycine (DHPG) resulted in a rapid 2.2 ± 0.5 fold increase in ERK1/2 phosphorylation within 1-5 min of agonist treatment (Fig. 1A). DHPG-stimulated ERK1/2 phosphorylation could be blocked by the mGluR1-specific antagonist LY367385 (Fig. 5B), but was unaffected by the treatment of cortical neurons with the mGluR5-specific antagonist MPEP (Fig. 5C). Therefore, ERK1/2 phosphorylation in primary mouse cortical neurons was selectively activated by mGluR1, despite the fact that both mGluR1 and mGluR5 subtypes are expressed in cortical tissue and cell culture [15].

**Figure 6:**
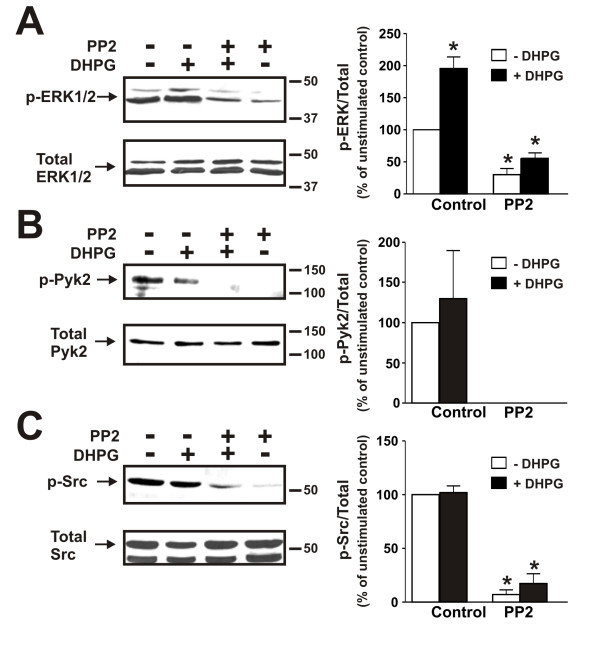
**Effect of the Src inhibitor PP2 on ERK1/2, Pyk2 and Src phosphorylation in cortical neurons**. **A) **Representative immunoblot showing the effect of treating primary mouse cortical neurons with and without 50 μM DHPG for 5 min and pretreated either with or without the Src inhibitor PP2 (10 μM) on ERK1/2 phosphorylation. **B) **Representative immunoblot showing the effect of treating primary mouse cortical neurons with and without 50 μM DHPG for 5 min and pretreated either with or without the Src inhibitor PP2 (10 μM) on Pyk2 (Y402) phosphorylation. **B) **Representative immunoblot showing the effect of treating primary mouse cortical neurons with and without 50 μM DHPG for 5 min and pretreated either with or without the Src inhibitor PP2 (10 μM) on Src (Y416) phosphorylation. Bar graph shows the mean ± SD of four independent experiments. * P < 0.05 versus untreated mGluR1a expressing cells.

To examine whether mGluR1a-mediated ERK1/2 phosphorylation was mediated by Src/Pyk2-mediated pathway, we pretreated primary mouse cortical neurons with and without the Src family kinase inhibitor PP2 (10 μM) for 60 min prior to treating the neurons with and without 50 μM DHPG. We found that, in the presence of the Src inhibitor, DHPG-stimulated ERK1/2 phosphorylation was effectively inhibited (Fig. 6A). We also observed that Pyk2 was phosphorylated in the absence of agonist and that DHPG treatment did not result in any further increase in Pyk2 phosphorylation (Fig. 6B). However, the treatment of the cortical neurons with PP2 completely suppressed Pyk2 (Y402) phosphorylation under both basal and agonist-stimulated conditions (Fig. 6B). Identical results were obtained when Src phosphorylation was examined (Fig. 6C). Thus, taken together the data indicated that Src activity was required for mGluR1-stimulated ERK1/2 phosphorylation and that suppression of Src activity resulted in an attenuation of Pyk2 phosphorylation.

**Figure 7:**
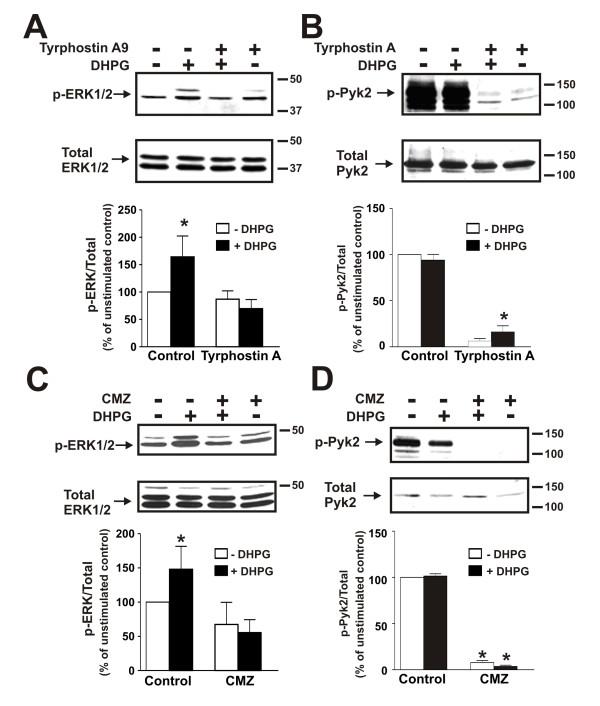
**Effect of tyrphostin A9 and calmidazolium chloride on ERK1/2 and Pyk2 phosphorylation in cortical neurons**. **A) **Representative immunoblot showing the effect of treating primary mouse cortical neurons with and without 50 μM DHPG for 5 min and pretreated either with or without tyrphostin A9 (10 μM) on ERK1/2 phosphorylation. Bar graph shows the mean ± SD of nine independent experiments. **B) **Representative immunoblot showing the effect of treating primary mouse cortical neurons with and without 50 μM DHPG for 5 min and pretreated either with or without tyrphostin A9 (10 μM) on Pyk2 (Y402) phosphorylation. Bar graph shows the mean ± SD of nine independent experiments. **C) **Representative immunoblot showing the effect of treating primary mouse cortical neurons with and without 50 μM DHPG for 5 min and pretreated either with or without 30 μM calmidazolum chloride (CMZ) on ERK1/2 phosphorylation. Bar graph shows the mean ± SD of five independent experiments.** D) **Representative immunoblot showing the effect of treating primary mouse cortical neurons with and without 50 μM DHPG for 5 min and pretreated either with or without 30 μM calmidazolum chloride (CMZ) on Pyk2 (Y402) phosphorylation. Bar graph shows the mean ± SD of five independent experiments. * P < 0.05 versus untreated mGluR1a expressing cells.

**Figure 8:**
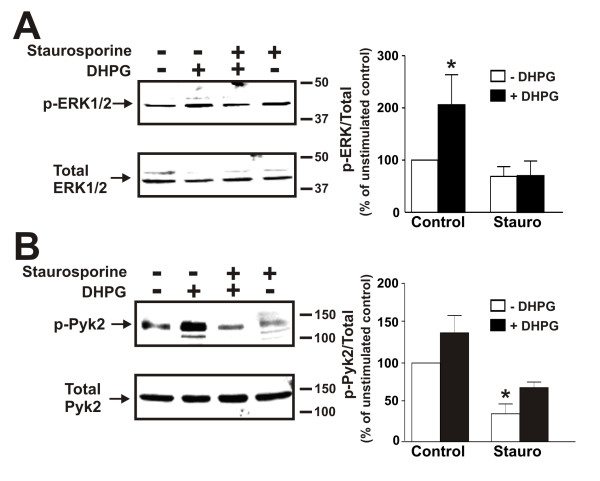
**Effect of Staurosporine on ERK1/2 and Pyk2 phosphorylation in cortical neurons**. **A) **Representative immunoblot showing the effect of treating primary mouse cortical neurons with and without 50 μM DHPG for 5 min and pretreated either with or without staurosporine (1 μM) on ERK1/2 phosphorylation. **B) **Representative immunoblot showing the effect of treating primary mouse cortical neurons with and without 50 μM DHPG for 5 min and pretreated either with or without staurosporine (1 μM) on Pyk2 (Y402) phosphorylation. Bar graph shows the mean ± SEM of five independent experiments. * P < 0.05 versus untreated mGluR1a expressing cells.

Tyrphostin A9 was previously demonstrated to be the most effective amongst an array of 51 tyrosine kinase inhibitors in antagonizing Pyk2 activity in neutrophils [23]. Therefore, to examine a role for Pyk2 in coupling mGluR1 to the activation of ERK1/2 phosphorylation, we utilized tyrphostin A9 to antagonize Pyk2 activity. The treatment of mouse primary neurons with 10 μM tyrphostin A9 for 10 min prior to agonist treatment effectively prevented ERK1/2 phosphorylation in response to 50 μM DHPG treatment (Fig. 7A). Consistent with an effect of tyrphostin A9 on Pyk2 activity, Pyk2 phosphorylation in either the absence or presence of DHPG was completely inhibited by the drug (Fig. 7B). Since tyrphostin A9 was not entirely selective for the antagonism of Pyk2 activity, we also examined the effect of a calmodulin antagonist calmidazolium chloride on mGluR1-stimulated ERK1/2 phosphorylation. Calmidazolium chloride was tested because Pyk2 was shown previously to be activated via a Ca^2+^-calmodulin dependent pathway in non-neuronal cells [24]. The pretreatment of cortical neurons with 30 μM calmidazolium chloride for 10 min also prevented DHPG-stimulated increases in ERK1/2 phosphorylation (Fig. 7C) and completely antagonized Pyk2 phosphorylation in either the absence or presence of DHPG (Fig. 7D). DHPG-mediated activation of ERK1/2 phosphorylation was also dependent upon PKC activation, as the treatment of cortical neurons with staurosporine blocked both ERK1/2 phosphorylation in response to agonist treatment (Fig. 8A) and reduced Pyk2 phosphorylation in either the absence or presence of DHPG (Fig. 8B).

**Figure 9:**
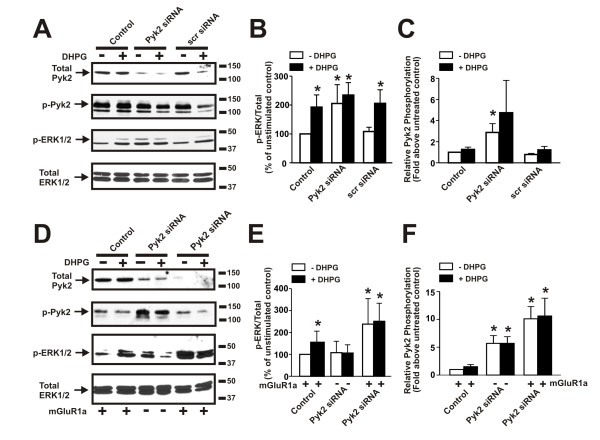
**Effect of Pyk2 siRNA treatment on ERK1/2 phosphorylation in cortical neurons and COS7 cells**. **A) **Representative immunoblots showing Pyk2 protein expression, ERK1/2 phosphorylation, and Pyk2 (Y402) phosphorylation in primary mouse cortical neurons treated with 100 nM Pyk2 siRNA and stimulated with and without 50 μM DHPG stimulation for 1 min. **B) **Bar graph shows the densitometric analysis of ERK1/2 phosphorylation in control, Pyk2 siRNA, and scrambled (Scr) siRNA treated cortical neurons and represents the mean ± SD of five independent experiments.** C) **Bar graph shows the densitometric analysis of Pyk2 (Y402) phosphorylation in control, Pyk2 siRNA, and scrambled (Scr) siRNA treated cortical neurons and represents the mean ± SD of five independent experiments.** D) **Representative immunoblots showing the effect of DHPG treatment (50 μM DHGP, 1 min) on Pyk2 protein expression, ERK1/2 phosphorylation, and Pyk2 (Y402) phosphorylation in COS7 cells expressing either FLAG-mGluR1 alone, treated with Pyk2 siRNA alone or expressing FLAG-mGluR1a and treated with Pyk2 siRNA. **E) **Bar graph shows the densitometric analysis of ERK1/2 phosphorylation in cells transfected with FLAG-mGluR1a alone, cells treated with Pyk2 siRNA alone, and cells transfected with FLAG-mGluR1a and treated with Pyk2 siRNA. **F) **Bar graph shows the densitometric analysis of Pyk2 (Y402) phosphorylation in cells transfected with FLAG-mGluR1a alone, cells treated with Pyk2 siRNA alone, and cells transfected with FLAG-mGluR1a and treated with Pyk2 siRNA. The graph represents the mean ± SD of four independent experiments. * P < 0.05 versus untreated mGluR1a expressing cells.

To further probe the role of Pyk2 in the activation of ERK1/2 by mGluR1, we utilized siRNA to knockdown endogenous Pyk2 expression in primary mouse cortical neurons. Pyk2 siRNA treatment of cortical neurons resulted in a 66 ± 5% decreases in Pyk2 expression (Fig. 9A). However, unlike what was expected the reduction in Pyk2 protein expression resulted in an increase in basal ERK1/2 phosphorylation to levels that were observed following mGluR1 activation with DHPG (Fig 9B). This increase in basal ERK/12 phosphorylation following Pyk2 siRNA treatment was also associated with an increase in the relative phosphorylation of the remaining Pyk2 protein expressed in the cortical neurons (Fig. 9C). To examine whether the increase in basal ERK1/2 phosphorylation was attributable to mGluR1 activity, we examined the effect of siRNA knockdown of Pyk2 in COS7 cells in the absence and presence of FLAG-mGluR1a expression. Pyk2 protein expression was reduced to 47 ± 5% in cells treated with Pyk2 siRNA alone when compared to cells treated with a scramble siRNA and in cells transfected with both FLAG-mGluR1a and Pyk2 siRNA, Pyk2 expression was further reduced to 16 ± 7% of control (Fig. 9D). In cells expressing FLAG-mGluR1a and treated with scrambled siRNA ERK1/2 phosphorylation was increased without a significant change in the extent of Pky2 phosphorylation in response DHPG treatment (Fig. 9E and 9F). In COS7 cells treated with Pyk2 siRNA alone (no FLAG-mGluR1a), Pyk2 phosphorylation was substantially increased, but no change in ERK1/2 phosphorylation was observed (Fig. 9E and 9F). However, the transfection of COS7 cells with both Pyk2 siRNA and FLAG-mGluR1a resulted in an even greater increase in Pyk2 phosphorylation and was associated with a substantial increase in basal ERK1/2 phosphorylation (Fig. 9E and 9F). Taken together, these observations indicated that the increased basal ERK1/2 phosphorylation observed following Pyk2 siRNA treatment was likely attributable to the basal activity of mGluR1.

**Figure 10:**
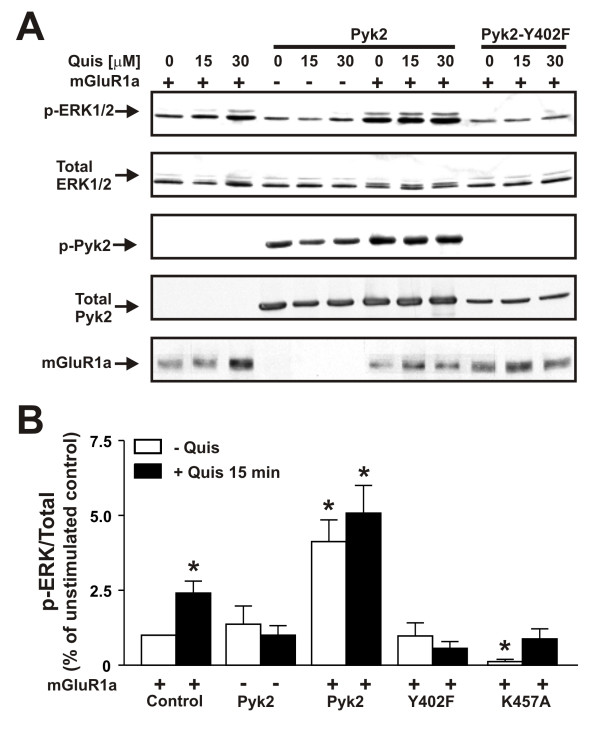
**Effect of Pyk2-Y402F and Pyk2 overexpression on mGluR1a-mediated ERK1/2 Phosphorylation**. ** A) **Representative immunoblots showing, ERK1/2 phosphorylation, ERK/12 protein expression, Pyk2 (Y402) phosphorylation, Pyk2 protein expression and FLAG-mGluR1a expression in cells transfected with and without pCDNA3.1 plasmd cDNAs encoding Flag-mGluR1a (5 μg) with and with 1 μg of pcDNA3.1 plasmid cDNA expressing either wild-type HA-Pyk2 or HA-Pyk2-Y402F. Cells were treated in the absence or presence of 50 μM quisqualate at 37°C. **B) **Bar graph shows the densitometric analysis of ERK1/2 phosphorylation in cells either expressing FLAG-mGluR1a alone, HA-Pyk2 alone or expressing FLAG-mGluR1a in the presence of HA-Pyk2, HA-Pyk2-Y402F and HA-Pyk2-K457A and is expressed as the mean ± SD of 4-8 different experiments. *P <0.05 compared to basal ERK1/2 phosphorylation levels in mGluR1a expressing cells.

To establish a direct role for Pyk2 in mGluR1a-mediated ERK1/2 phosphorylation, we examined the effect of overexpressing wild-type Pyk2, a dominant negative Pyk2 (Y402F) mutant and a catalytically inactive Pyk2 (K457A) mutant on FLAG-mGluR1 signaling in HEK293 cells. We found that FLAG-mGluR1a activation with 100 μM quisqualate resulted in a 2.4 ± 0.4 fold increase in ERK1/2 phosphorylation in the absence of Pyk2 overexpression (Fig. 10A and 10B). Overexpression of HA-Pyk2 alone (no FLAG-mGluR1a) had no effect on either basal or quisqualate-stimulated ERK1/2 phosphorylation in the absence of FLAG-mGluR1a expression. However, overexpression of HA-Pyk2 with FLAG-mGluR1a resulted in a 4.1 ± 0.7 fold increase in basal ERK1/2 expression, when compared to basal ERK1/2 phosphorylation in cells expressing FLAG-mGluR1a alone (Fig. 10A and 10B). Furthermore, agonist stimulation with quisqualate did not result in a substantial increase in ERK1/2 phosphorylation following the overexpression of HA-Pyk2 (Fig. 10A and 10B). In contrast, overexpression of either a dominant-negative Pyk2-Y402F mutant or catalytically inactive Pyk2-K457A mutant prevented FLAG-mGluR1a-stimulated increases in ERK1/2 phosphorylation (Fig. 10A and 10B). The overexpression of Pyk2-K457A also resulted in reduced basal ERK1/2 phosphorylation (Fig. 10A and 10B). Taken together, these data indicated that in addition to contributing to the uncoupling of mGluR1 from Gα_q/11_, Pyk2 was required for mGluR1-dependent activation of ERK1/2 signaling.

## Discussion

In the present study, we provide evidence that the non-receptor tyrosine kinase Pyk2 interacts with and regulates mGluR1a signaling and desensitization. Pyk2 associates with the second intracellular loop domain and the carboxyl-terminal tail domains of mGluR1a, and similar to what has been described for GRK2, optineurin and CAIN, Pyk2 functions to disrupt Gα_q/11 _interactions with the receptor resulting in the attenuation of IP formation [9,16,17]. However, Pyk2 also facilitates mGluR1a-mediated activation of the ERK1/2 pathway, an effect that also requires the activity of Src, calmodulin and PKC. Thus, we describe a new mechanism by which Pyk2 attenuates mGluR1a by disrupting mGluR1a/Gα_q/11 _interactions, while simultaneously coupling the receptor to the activation of the mitogen-activated protein kinase (MAPK) signaling pathway.

Pyk2 is a non-receptor tyrosine kinase that is known to be activated by stimuli that either increase intracellular Ca^2+ ^concentrations or activate PKC in response to stress signals, such as UV light, hyperosmotic shock and tumor necrosis factor-α [25]. Pyk2 has also been shown to be involved in G protein-coupled-receptor signaling. For example, Felsch *et al. *[26] reported that Pyk2 phosphorylates the potassium channel Kv1.2 in response to the activation of m1 muscarinic acetylcholine receptor. In vascular smooth muscle cells, angiotensin II stimulates the association of Janus kinase 2 with the angiotensin II type1 receptor via a mechanism that involves increases in intracellular Ca^2+ ^concentrations and the activation of both PKCδ and Pyk2 [27]. Similarly, Heidinger *et al. *[21] showed that Pyk2 is involved in the mGluR1-mediated phopshorylation of NR2A/B subunits of the NMDA receptor in cortical neurons. Moreover, in HEK293 cells both the α_1B_- or α_2A_-adrenergic receptors stimulate ERK1/2 phosphorylation via a Ca^2+^-, calmodulin-, Src- and Pyk2-dependent pathway [24]. In the present study, we provide evidence that Pyk2 interacts with mGluR1a and plays a role in of the mGluR1a-dependent activation of ERK1/2 phosphorylation via a mechanism that also requires calmodulin, Src, and PKC activity. Moreover, Pyk2 is associated with mGluR1a in the absence of agonist treatment and the mGluR1a/Pyk2 protein complex dissociates in response to quisqualate treatment for 20 min. The physiological consequence of this dissociation is unclear, but it is possible that the loss of Pyk2 from the receptor may occur as the result GRK2 binding to the receptor, which like mGluR5 might be required for agonist-stimulated mGluR1a endocytosis [33]. Consistent with this hypothesis and the observation that Pyk2 dissociates from the receptor in response to agonist activation, we do not observe Pyk2 internalization with mGluR1a.

Pyk2 has previously been demonstrated to be autophosphorylated on Tyr402 in response to the activation of the G protein-coupled lysophosphatidic acid receptor resulting in the creation of a docking site for the Src SH2 domain [28]. The recruitment of Src to Pyk2 subsequently leads to Pyk2 phosphorylation at both Tyr579 and Tyr580 and results in enhanced Pyk2 kinase activity [28-30]. The overexpression of Pyk2 is also reported to result in increased basal ERK1/2 phosphorylation [28,29,31]. However, we find that in HEK293 cells basal ERK1/2 phosphorylation is increased only when mGluR1a is overexpressed with Pyk2. Thus, despite the fact that Pyk2 reduces basal mGluR1a G protein coupling, Pyk2 overexpression results in an increase in basal ERK1/2 phosphorylation. In contrast, mGluR1a-mediated ERK1/2 phosphorylation is reduced following the expression of dominant-negative Pyk2-Y402F and catalytically inactive Pyk2-K457A mutants. Thus, we conclude that Pyk2 is essential for ERK1/2 phosphorylation in response to mGluR1a activation.

Both mGluR1 and mGluR5 are expressed in cortical tissue and primary cortical neurons can be positively stained for the expression of both mGluR1a and mGluR5 proteins [15]. However, in primary mouse cortical neurons ERK1/2 phosphorylation is selectively activated by mGluR1, as ERK1/2 phosphorylation in response to DHPG is not inhibited by a mGluR5-specific antagonist. This is similar to what was observed by Heidinger and colleagues [21], who demonstrated that the phosphorylation of NMDA receptors by Pyk2 is selectively mediated by the activation of mGluR1 and not mGluR5 in cortical neurons. It is likely that mGluR5 will also activate ERK1/2 phosphorylation in other neuronal cell types for several reasons. First, Pyk2 can be co-immunoprecipitated from rat brain with mGluR5. Second, mGluR5-mediated nociception in the spinal cord involves the activation of ERK1/2 and we have recently demonstrated that mGluR5-dependent ERK1/2 phosphorylation is increased in mutant huntingtin protein knockin mice [32,33]. Finally, MacDonald and coworkers have demonstrated that mGluR5-dependent activation of Pyk2 stimulates NMDA and AMPA receptor currents in hippocampal neurons [34].

It has previously been reported that Group I mGluRs increase the extent of Pyk2 phosphorylation in mouse neuronal cortical cultures via a mechanism that is both calmodulin- and Src-dependent, but that is independent of PKC activity [21]. We find that Pyk2-mediated activation of ERK1/2 phosphorylation is both calmodulin- and Src-dependent, but it also involves a mechanism that requires PKC activation. Calmodulin binds to both mGluR1 and mGluR5 in a Ca^2+^-dependent manner and can be regulated by PKC-mediated phosphorylation of the receptor within the calmodulin binding domain [35-37]. The identification of Pyk2 and CaM as Group I mGluR interacting proteins suggests the possibility that Pyk2, Src and calmodulin may exist as a preformed complex that is scaffolded on the intracellular face of mGluR1. Thus, in response to PKC-mediated phosphorylation of mGluR1a, the complex may be released from the receptor to activate ERK1/2 phosphorylation.

We also find that siRNA treatment of cortical neurons results in a significant reduction in Pyk2 protein expression, but unexpectedly this also results in increased basal ERK1/2 phosphorylation. The increase in ERK1/2 phosphorylation is associated with increased phosphorylation of the remaining fraction of Pyk2 protein expressed in the neuronal cultures. The depletion of Pyk2 expression in COS7 cells also leads to increased basal Pyk2 phosphorylation, but mGluR1a expression is required for this Pyk2-mediated increase in ERK/1/2 phosphorylation. The mechanism by which a loss of Pyk2 expression leads to alterations in the phosphorylation of the residual pool of Pyk2 and increased ERK1/2 phosphorylation is unclear. However, it appears to require mGluR1 expression suggesting that constitutive mGluR1 activity underlies the phenomenon and that Pyk2 plays a role in regulating basal mGluR1 activity.

In summary, we show here that Pyk2 interacts with Group I mGluR1a via the intracellular loop2 and the carboxyl-terminal tail domains of mGluR1a and functions to uncouple the receptor from Gα_q/11 _protein, while facilitating ERK1/2 phosphorylation. The direct association of Pyk2 with mGluR1a appears to coordinate the formation of a protein complex that includes Src bound to Pyk2 and potentially calmodulin bound to the carboxyl-terminal tail of the receptor. The formation of this complex may be important for the spatial temporal regulation of Pyk2 activity at post-synaptic densities that is expected to be required for the efficient regulation of NMDA and AMPA receptor function as well as the regulation of other ERK1/2-dependent activities in synaptic transmission. The observation that several signaling proteins, such as Pyk2, optineurin CAIN and GRK2 regulate mGluR signaling via their interaction with the interface provided by the second intracellular loop suggests that peptides which mimic this interface may be useful for the modulation of Group I mGluR signaling.

## Abbreviations

ANOVA: analysis of variance; DIV: days *in vitro*; CAIN: calcineurin inhibitor protein; COS7: African green monkey cells; CMZ: calmidazolum chloride; DHPG: (S)-3,5-dihydroxylphenylglycine; ECL: enhanced chemiluminescence; ERK: extracellular signal-regulated kinase; GPCR: G protein-coupled receptor; GRK: G protein-coupled receptor kinase; GST: glutathione S transferase; HA: hemagglutinin; HEK293 cell: Human embryonic kidney cell; IP: inositol phosphate; InsP_3_: inositol-1,4,5-triphosphate; mGluR: metabotropic glutamate receptor; MAPK: mitogen-activated protein kinase; MPEP: 2-methyl-6-(phenylethynyl)-pyridine; NMDAR: N-methyl-D-aspartate receptor; PKC: protein kinase C; Pyk2: proline-rich tyrosine kinase 2.

## Competing interests

The authors declare that they have no competing interests.

## Authors' contributions

AAN, MP, LTF and FMR performed the experiments included in the manuscript. LBD and TC provided technical assistance with cell cultures and confocal microscopy. SSGF conceived of the experiments and wrote the manuscript. All of the authors have read the manuscript.
